# Decoding Huntington’s disease: a global survey on symptoms and genetic testing practices

**DOI:** 10.1007/s00406-025-02042-8

**Published:** 2025-06-25

**Authors:** C.A.M Koriath, C. Kurz, S. Mead, E.J. Wild, S.J. Tabrizi

**Affiliations:** 1 LMU Department of Psychiatry and Psychotherapy, University Hospital Munich, Munich, Germany; 2 MRC Prion Unit, UCL Institute of Prion Disease, Courtauld Building, London, W1W 7FF UK; 3 Huntington’s Disease Centre, Department of Neurodegenerative Disease, UCL Queen Square Institute of Neurology, London, UK

**Keywords:** Huntington’s disease, HD, HD phenocopy, HD-like, Neurodegenerative disease, Genetic testing

## Abstract

**Background:**

Huntington’s disease (HD) is a fatal, autosomal dominant neurodegenerative disorder caused by a CAG trinucleotide repeat expansion in the *HTT* gene. While chorea is the hallmark motor symptom, HD presents with diverse psychiatric and cognitive manifestations that usually precede motor onset.

**Methods:**

A 10-question online survey was distributed to 130 neurologists and neuro-geneticists from the European Huntington’s Disease Network (EHDN) to identify clinical symptoms considered pathognonomic of HD and criteria for genetic testing. Responses from 52 specialists were anonymized and analysed using Microsoft Excel and SPSS 26.

**Results:**

Respondents, averaging 18.4 years of experience, universally identified chorea as indicative of HD, alongside cognitive slowing, irritability, and gait abnormalities. Symptoms like neuropathy, limb weakness, and tremor were deemed inconsistent with HD. Notably, 19% of experts reported that ancillary symptoms would not deter them from recommending testing if a primary HD symptom was present. Without a family history, only chorea with or without additional symptoms was deemed sufficient for testing.

**Discussion:**

The findings highlight the complexity of diagnosing HD, the importance of considering subtle psychiatric and cognitive symptoms, and the need for comprehensive patient counselling. Advances in genetic testing and therapeutic trials targeting the molecular root of HD offer hope for curative treatments.

**Conclusion:**

This study underscores the growing recognition of HD’s pleiotropy, the ethical considerations in testing, and the importance of clinical vigilance as patients may often first present in a non-neurological setting.

**Supplementary Information:**

The online version contains supplementary material available at 10.1007/s00406-025-02042-8.

## Introduction

Huntington’s disease (HD), which is typically defined by a progressive triad of movement, cognitive, and psychiatric symptoms^1^, is the commonest fatal, autosomal dominant, adult onset neurodegenerative disorders with a prevalence of at least 12.4 per 100,000 people [1, 2]. It is caused by a CAG trinucleotide repeat expansion in the huntingtin gene on chromosome 4; the number of repeats inversely correlates with age at onset (AAO) [3]. Chorea is considered the most well recognized feature of HD, but motor symptoms can range from hyperkinetic to hypokinetic. In addition, HD patients may generally experience a diverse range of symptoms, including cognitive and behavioural impairment, usually preceding the onset of unequivocal motor extrapyramidal symptoms [4, 5]. Both motor and non-motor manifestations are caused by disruption of striatal function through the direct and indirect pathways [1]. The heterogeneous presentation may render the clinical diagnosis difficult and explain why the return rate of negative HD tests has increased to approx. 20% [2]. We therefore undertook a survey of expert opinion and practice with regards to what clinical symptoms they consider to be typical or non-typical for HD and what symptoms should warrant HD testing.

## Methods

We invited 130 neurologists and neuro-geneticists from the European Huntington’s Disease Network (EHDN) to participate in a 10-question online survey. Clinicians were contacted via email, with at least one from each EHDN site across Europe and the USA. Questions referred to “HD and HD phenocopy patients”, HD phenocopy (HDPC) patients being patients who are tested for HD but receive a negative test result. Responses were anonymised and analysed using Microsoft Excel and SPSS 26. In order to establish which symptoms informed the decision of neurologists, psychiatrists, and geneticists with experience in HD to order genetic testing, we compared symptom responsesusing Bonferroni-corrected Chi-Square tests (Table 1).

**Table 1:** Symptoms expected to be typical for HD or not

Characteristic or Symptom		HD	HD-like			p-value (Bonferroni corrected, Chi-Square Test))	
**A) Symptoms considered typical for HD**							
Chorea		52 (100%)	1 (1.9%)			6.70*10^–29^*	
Irritability		34 (65.4%)	0 (0%)			2.93*10^–14^*	
Dysexecutive Syndrome		31 (59.6%)	0 (0%)			1.37*10^–12^*	
Falls		30 (57.7%)	2 (3.8%)			1.15*10^− 9^*	
Gait abnormality		30 (57.7%)	2 (3.8%)			1.15*10^− 9^*	
Apathy		28 (53.8%)	0 (0%)			4.7599*10^–11^*	
Cognitive Slowing		25 (48.1%)	1 (1.9%)			2.25*10^− 08^*	
Dystonia		20 (38.5%)	1 (1.9%)			0.000003*	
Depression		20 (38.5%)	3 (5.8%)			0.000089*	
Anxiety		17 (32.7%)	2 (3.8%)			0.0002*	
Disinhibition		16 (30.8%)	4 (7.7%)			0.005	
Loss of Empathy		13 (25%)	1 (1.9%)			0.001*	
Agitation		12 (23.1%)	1 (1.9%)			0.001*	
Obsessive Behaviour		12 (23.1%)	1 (1.9%)			0.002	
Weight Loss		11 (21.2%)	0 (0%)			0.001*	
Dysarthria		12 (23.1%)	2 (3.8%)			0.008	
Dysphagia /Choking		11 (21.2%)	1 (1.9%)			0.004	
Rigidity		9 (17.3%)	5 (9.6%)			n.s.	
Delusions		4 (7.7%)	1 (1.9%)			n.s.	
Memory Loss		9 (17.3%)	7 (13.5%)			n.s.	
Insomnia		4 (7.7%)	3 (5.8%)			n.s.	
Paranoia		5 (9.6%)	0 (0%)			n.s.	
Disorientation/ Navigation		5 (9.6%)	5 (9.6%)			n.s.	
**B) Symptoms not considered typical for HD**							
Motor Neuropathy	0 (0%)			38 (73.1%)	9.78*10^− 17^*		
Sensory Neuropathy	0 (0%)			38 (73.1%)	9.78*10^− 17^*		
Limb weakness	0 (0%)			30 (57.7%)	4.6096*10^− 12^*		
Pain	0 (0%)			28 (53.8%)	4.76*10^− 11^*		
Tremor	0 (0%)			27 (51.9%)	1.29*10^− 9^*		
Ataxia	6 (11.5%)			22 (42.3%)	0.001		
Hallucinations	2 (3.8%)			13 (25%)	0.004		
Hypersomnia	0 (0%)			7 (13.5%)	0.013		
Sweet Tooth/Change in dietary habits	0 (0%)			6 (11.5%)	0.027		

P-values marked * remain significant at 0.05 after correcting for multiple testing using the Bonferroni method

## Results

Fifty-two specialists responded, with an average of 18.4 years of experience (range: 3–36 years) and an average caseload of 17 HD patients per month (range: 1–50). Respondents were from the UK (12), continental Europe (19), and the USA (21). Chorea was universally regarded as indicative of HD (100%), along with cognitive slowing (30.8%), executive dysfunction (30.8%), irritability (30.8%), gait abnormalities/falls (28.9%), and dystonia (25.0%). Symptoms like neuropathy (74.5%), limb weakness (54.9%), ataxia (35.3%), pain (33.3%), tremor (27.5%), and hallucinations (21.6%) were more commonly associated with other syndromes (Table S-1, Q5 and Q7). Despite this, 19% (10/52) of HD experts indicated that no ancillary symptom would dissuade them from expecting a positive HD test if a primary HD symptom was present (Table S-1, Q10). Chi-square analysis identified significant expected symptom differences between HD and HD phenocopy syndromes, with chorea and dystonia, depression and irritability, cognitive slowing, apathy, executive dysfunction, gait abnormalities, and falls expected to be more common in HD, while neuropathy, limb weakness, pain, falls, and tremor considered to be unlikely to be consistent with HD. In the absence of a positive family history, only chorea alone or in combination with additional symptoms was deemed sufficient to refer patients for genetic testing (Table S-1, Q8).

## Discussion

**Fig. 1:**
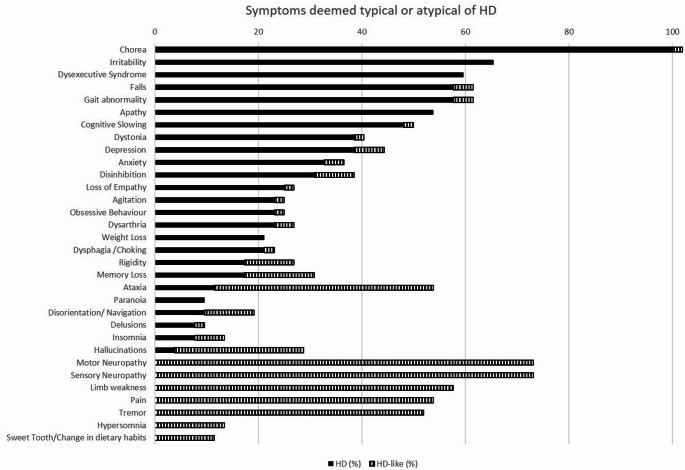
Symptoms deemed typical or atypical for HD. This figure shows the symptoms HD experts deemed typical or atypical for HD, with answers for HD shown in black and for HD-like in stripes. Answers are shown as a percentage of HD experts answering positively for each symptom and each category

Since the publication of the HTT expansion test in 1993, the recognized phenotype of HD has broadened. This survey aimed to establish what HD experts consider typical and when they might order a genetic test, considering clinical presentation and ethical implications. The results highlight typical HD symptoms—chorea, dystonia, cognitive slowing, apathy, and depression—while emphasising the condition’s heterogeneity, as well as subtle initial cognitive and psychiatric symptoms. Insomnia, dysarthria, and dysphagia are common in HD, while progression usually leads to gait disturbances and falls. Interestingly, 20% of respondents maintained their expectation of a positive HD test, even when symptoms typically associated with other syndromes were present, provided a primary HD symptom was evident. Indeed, this is consistent with our recent study demonstrating that HD and HDPC patients tested for HD, whose test result is negative are clinically indistinguishable [2]However, the emphasis given to chorea was misplaced as the rate of chorea was similar in both the HD and HDPC cohorts [2]. Consistent with HD experts expectations, the lack of any relevant family history of cognitive, psychiatric or motor symptoms currently appears to most strongly indicate a likely negative HD test [2]. Rather than being a result of alleged indiscriminate genetic testing, the increase in negative HD test results therefore reveals the increasing acceptance of HD pleiotropy (Fig. 1).

Genetic testing requires informed consent, which can become complex when patients have cognitive impairments [6]. Predictive testing for neurodegenerative diseases, traditionally managed by clinical geneticists, follows established protocols but can be emotionally taxing. Consequently, uptake rates are typically low, averaging around 20–25% for conditions like HD and prion disease [6, 7]. However, the advent of successful treatment trials for genetic neurological disorders [8] is likely to alter these trends. The number of CAG repeats in the *HTT* gene inversely correlates with AAO; however, this correlation is non-linear and accounts for only about half of the observed variation [1]. The toxicity associated with the CAG repeat expansion arises from multiple factors, including full-length expanded huntingtin, N-terminal huntingtin fragments, aberrant splicing of intron 1 of the *HTT* gene, and somatic expansion of the CAG repeat [8]. AAO and disease progression in HD are influenced by genetic loci within DNA repair genes, such as *FAN1* [9, 10], whose proteins influence somatic CAG repeat expansion [11]. Although the phase 3 efficacy trial for Tominersen, an antisense oligonucleotide, was halted due to poorer clinical outcomes and increased serious adverse events in the high-dose group [8], targeting *HTT* and its genetic modifiers remains promising. These approaches aim to address the molecular root of HD, potentially paving the way for curative treatments for HD and other expansion disorders in the future [12]. The study is limited by the subjective nature of the survey and low or zero cell counts in some symptom categories, which may affect the robustness of the Chi-square tests. The approach was retained for analytic consistency, and the study remains representative of usual clinical practice given the respondents’ expertise.

## Conclusion

This study underscores the complexity of diagnosing HD and genetic testing, highlighting its diverse presentation and ethical challenges. While chorea remains a hallmark feature, cognitive and psychiatric symptoms significantly impact patients’ quality of life and usually precede motor symptoms. Clinicians should therefore remain vigilant for potential cases of HD, even when the full triad of symptoms is not present, and should feel confident in referring patients for genetic testing when warranted. Expert responses emphasize the need for informed consent and comprehensive patient counselling to navigate testing decisions. Advances in understanding HD’s molecular basis and emerging therapeutic trials hold promise for improving care and potentially delivering curative treatments in the future.

## Electronic supplementary material

Below is the link to the electronic supplementary material.

Supplementary Material 1
