# Supplementary material for: Decoding Huntington’s disease: a global survey on symptoms and genetic testing practices
Source: Eur Arch Psychiatry Clin Neurosci. 2025 Jun 25;275(8):2557–61. doi: 10.1007/s00406-025-02042-8 (PMC12638385; doi:10.1007/s00406-025-02042-8)
Supplement: Supplementary file 1 — Supplementary Material 1 [file 406_2025_2042_MOESM1_ESM.docx]

**Table S-1: Survey questions sent to HD experts**

| **Q1. What is your professional background? Please state your specialty.** | | | |
| --- | --- | --- | --- |
| Neurology | 36 / 70.6% | Movement Disorders | 25 / 49% |
| Neurogenetics | 11 / 21.6% | Psychiatry | 5 / 9.8% |
| Clinical Genetics | 1 / 2% |  |  |
| Answered | 51 |  |  |
| **Q2. How many years have you been working with HD and HD phenocopy patients?** | | |  |
| 3 years | 1 / 1.9% | 17 years | 1 / 1.9% |
| 4 years | 1 / 1.9% | 19 years | 2 / 3.8% |
| 6 years | 2 / 3.8% | 20 years | 9 / 17.3% |
| 7 years | 1 / 1.9% | 22 years | 1 / 1.9% |
| 8 years | 2 / 3.8% | 23 years | 2 / 3.8% |
| 10 years | 4 / 7.7% | 25 years | 4 / 7.7% |
| 11 years | 3 / 5.8% | 28 years | 1 / 1.9% |
| 12 years | 1 / 1.9% | 30 years | 6 / 11.5% |
| 13 years | 1 / 1.9% | 31 years | 1 / 1.9% |
| 14 years | 2 / 3.8% | 34 years | 1 / 1.9% |
| 15 years | 5 / 9.6% | 36 years | 1 / 1.9% |
| Answered | 52 |  |  |
| **Q3. How many HD and/or HD phenocopy patients do you typically see in a month?** | | |  |
| 1 patient | 3 / 5.8% | 18 patients | 2 / 3.8% |
| 2 patients | 1 / 1.9% | 20 patients | 5 / 9.6% |
| 4 patients | 2 / 3.8% | 25 patients | 4 / 7.7% |
| 5 patients | 2 / 3.8% | 30 patients | 2 / 3.8% |
| 6 patients | 2 / 3.8% | 35 patients | 1 / 1.9% |
| 8 patients | 3 / 5.8% | 40 patients | 4 / 7.7% |
| 10 patients | 12 / 23.1% | 45 patients | 1 / 1.9% |
| 12 patients | 1 / 1.9% | 50 patients | 1 / 1.9% |
| 15 patients | 4 / 7.7% |  |  |
| Answered | 52 |  |  |
| **Q4. In a patient with a compatible syndrome and a possibly positive family history (but without a genetic diagnosis in the family), what symptom or symptoms would make you most strongly consider an HD test?** | | | |
| Chorea | 52 / 100% | Agitation | 12 / 23.1% |
| Dystonia | 20 / 38.5% | Apathy | 28 / 53.9% |
| Rigidity | 9 / 17.3% | Anxiety | 17 / 32.7% |
| Gait abnormalities / Falls | 30 / 57.7% | Depression | 20 / 38.5% |
| Ataxia | 6 / 11.5% | Irritability | 34 / 65.4% |
| Tremor | 0 / 0% | Disinhibition | 16 / 30.8% |
| Dysarthria | 12 / 23.1% | Obsessive behaviour | 12 / 23.1% |
| Dysphagia / Choking | 11 / 21.2% | Paranoia | 5 / 9.6% |
| Memory loss | 9 / 17.3% | Delusions | 4 / 7.7% |
| Disorientation / Navigational difficulties | 5 / 9.6% | Hallucinations | 2 / 3.9% |
| Cognitive slowing | 25 / 48.1% | Change in dietary habits | 0 / 0% |
| Dysexecutive syndrome | 31 / 59.6% | Limb weakness | 0 / 0% |
| Loss of empathy | 13 / 25% | Weight loss | 11 / 21.2% |
| Insomnia | 4 / 7.7% | Pain | 0 / 0% |
| Hypersomnia | 0 / 0% | Neuropathy (sensory / motor) | 0 / 0% |
| Answered | 52 |  |  |
| **Q5. Based on Q4, please tick only your top 3 symptom which would make you consider an HD test.** | | | |
| Chorea | 52 / 100% | Agitation | 0 / 0% |
| Dystonia | 13 / 25% | Apathy | 6 / 11.5% |
| Rigidity | 1 / 1.9% | Anxiety | 2 / 3.9% |
| Gait abnormalities / Falls | 15 / 28.9% | Depression | 2 / 3.9% |
| Ataxia | 2 / 3.9% | Irritability | 16 / 30.8% |
| Tremor | 0 / 0% | Disinhibition | 6 / 11.5% |
| Dysarthria | 1 / 1.9% | Obsessive behaviour | 0 / 0% |
| Dysphagia / Choking | 2 / 3.9% | Paranoia | 1 / 1.9% |
| Memory loss | 3 / 5.8% | Delusions | 0 / 0% |
| Disorientation / Navigational difficulties | 0 / 0% | Hallucinations | 0 / 0% |
| Cognitive slowing | 16 / 30.8% | Change in dietary habits | 0 / 0% |
| Dysexecutive syndrome | 16 / 30.8% | Limb weakness | 0 / 0% |
| Loss of empathy | 1 / 1.9% | Weight loss | 1 / 1.9% |
| Insomnia | 1 / 1.9% | Pain | 0 / 0% |
| Hypersomnia | 0 / 0% | Neuropathy (sensory / motor) | 0 / 0% |
| Answered | 52 |  |  |
| **Q6. In such a patient, is there a clinical symptom (or symptoms) that, if present, would make you less inclined to order an HD test, possibly because that symptom is making another diagnosis more likely in your mind?** | | | |
| Chorea | 1 / 2% | Agitation | 1 / 2% |
| Dystonia | 1 / 2% | Apathy | 0 / 0% |
| Rigidity | 5 / 9.8% | Anxiety | 2 / 3.9% |
| Gait abnormalities / Falls | 2 / 3.9% | Depression | 3 / 5.9% |
| Ataxia | 22 / 43.1% | Irritability | 0 / 0% |
| Tremor | 25 / 49% | Disinhibition | 4 / 7.8% |
| Dysarthria | 2 / 3.9% | Obsessive behaviour | 1 / 2% |
| Dysphagia / Choking | 1 / 2% | Paranoia | 0 / 0% |
| Memory loss | 7 / 13.7% | Delusions | 0 / 0% |
| Disorientation / Navigational difficulties | 5 / 9.8% | Hallucinations | 13 / 25.5% |
| Cognitive slowing | 1 / 2% | Change in dietary habits | 6 / 11.8% |
| Dysexecutive syndrome | 0 / 0% | Limb weakness | 30 / 58.8% |
| Loss of empathy | 1 / 2% | Weight loss | 0 / 0% |
| Insomnia | 3 / 5.9% | Pain | 28 / 54.9% |
| Hypersomnia | 7 / 13.7% | Neuropathy (sensory / motor) | 38 / 74.5% |
| Answered | 51 |  |  |
| **Q7. Based on Q6, please tick only your 3 top symptom choices that would make you less inclined to order an HD test.** | | | |
| Chorea | 0 / 0% | Agitation | 0 / 0% |
| Dystonia | 0 / 0% | Apathy | 0 / 0% |
| Rigidity | 1 / 2% | Anxiety | 1 / 2% |
| Gait abnormalities / Falls | 0 / 0% | Depression | 1 / 2% |
| Ataxia | 18 / 35.3% | Irritability | 0 / 0% |
| Tremor | 14 / 27.5% | Disinhibition | 1 / 2% |
| Dysarthria | 0 / 0% | Obsessive behaviour | 0 / 0% |
| Dysphagia / Choking | 1 / 2% | Paranoia | 0 / 0% |
| Memory loss | 4 / 7.8% | Delusions | 0 / 0% |
| Disorientation / Navigational difficulties | 3 / 5.9% | Hallucinations | 11 / 21.6% |
| Cognitive slowing | 0 / 0% | Change in dietary habits | 6 / 11.8% |
| Dysexecutive syndrome | 0 / 0% | Limb weakness | 28 / 54.9% |
| Loss of empathy | 0 / 0% | Weight loss | 1 / 2% |
| Insomnia | 1 / 2% | Pain | 17 / 33.3% |
| Hypersomnia | 3 / 5.9% | Neuropathy (sensory / motor) | 38 / 74.5% |
| Answered | 51 |  |  |
| **Q8. What combination of symptoms would be sufficient for you to consider HD and test for it?** | | |  |
| Chorea - cognitive problems - anxiety - with family history (one affected parent or grandparent) | | | 48 / 92.3% |
| Chorea - cognitive problems - anxiety - no family history | | | 43 / 82.7% |
| Dystonia - cognitive problems - anxiety - with family history (one affected parent or grandparent) | | | 36 / 69.2% |
| Dystonia - cognitive problems - anxiety - no family history | | | 19 / 36.5% |
| Rigidity- cognitive problems - apathy- with family history (one affected parent or grandparent) | | | 31 / 59.6% |
| Rigidity- cognitive problems - apathy- no family history | | | 11 / 21.2% |
| Chorea - with family history (one affected parent or grandparent) | | | 46 / 88.5% |
| Chorea - no family history | | | 36 / 69.2% |
| Dystonia - with family history (one affected parent or grandparent) | | | 27 / 51.9% |
| Dystonia - no family history | | | 3 / 5.8% |
| Rigidity - with family history (one affected parent or grandparent) | | | 25 / 48.1% |
| Rigidity - no family history | | | 2 / 3.9% |
| Dysexecutive syndrome - with family history (one affected parent or grandparent) | | | 29 / 55.8% |
| Dysexecutive syndrome - no family history | | | 2 / 3.9% |
| Irritability - cognitive slowing - with family history (one affected parent or grandparent) | | | 34 / 65.4% |
| Irritability - cognitive slowing - no family history | | | 8 / 15.4% |
| Dysexecutive syndrome - depression - with family history (one affected parent or grandparent) | | | 30 / 57.7% |
| Dysexecutive syndrome - depression - no family history | | | 2 / 3.9% |
| Disinhibition - with family history (one affected parent or grandparent) | | | 20 / 38.5% |
| Disinhibition - no family history | | | 1 / 1.9% |
| Answered |  |  | 52 |
| **Q9. Are there any symptoms that, if present, would make you expect that an HD test will come back negative, despite the obvious need to test for (and exclude) HD?** | | | |
| Chorea | 0 / 0% | Agitation | 0 / 0% |
| Dystonia | 1 / 1.9% | Apathy | 0 / 0% |
| Rigidity | 0 / 0% | Anxiety | 1 / 1.9% |
| Gait abnormalities / Falls | 0 / 0% | Depression | 1 / 1.9% |
| Ataxia | 11 / 21.2% | Irritability | 0 / 0% |
| Tremor | 13 / 25% | Disinhibition | 0 / 0% |
| Dysarthria | 1 / 1.9% | Obsessive behaviour | 0 / 0% |
| Dysphagia / Choking | 0 / 0% | Paranoia | 1 / 1.9% |
| Memory loss | 4 / 7.7% | Delusions | 1 / 1.9% |
| Disorientation / Navigational difficulties | 2 / 3.9% | Hallucinations | 6 / 11.5% |
| Cognitive slowing | 0 / 0% | Change in dietary habits | 3 / 5.8% |
| Dysexecutive syndrome | 1 / 1.9% | Limb weakness | 24 / 46.2% |
| Loss of empathy | 0 / 0% | Weight loss | 1 / 1.9% |
| Insomnia | 1 / 1.9% | Pain | 21 / 40.4% |
| Hypersomnia | 9 / 17.3% | Neuropathy (sensory / motor) | 32 / 61.5% |
| None of these | 12 / 23.1% |  |  |
| Answered | 52 |  |  |
| **Q10. Based on Q9, which top 3 symptoms would make you expect that an HD test will come back negative, despite the obvious need to test for it?** | | | |
| Chorea | 0 / 0% | Agitation | 0 / 0% |
| Dystonia | 1 / 1.9% | Apathy | 0 / 0% |
| Rigidity | 0 / 0% | Anxiety | 1 / 1.9% |
| Gait abnormalities / Falls | 0 / 0% | Depression | 1 / 1.9% |
| Ataxia | 8 / 15.4% | Irritability | 0 / 0% |
| Tremor | 10 / 19.2% | Disinhibition | 0 / 0% |
| Dysarthria | 0 / 0% | Obsessive behaviour | 0 / 0% |
| Dysphagia / Choking | 0 / 0% | Paranoia | 1 / 1.9% |
| Memory loss | 2 / 3.9% | Delusions | 1 / 1.9% |
| Disorientation / Navigational difficulties | 2 / 3.9% | Hallucinations | 4 / 7.7% |
| Cognitive slowing | 0 / 0% | Change in dietary habits | 3 / 5.8% |
| Dysexecutive syndrome | 1 / 1.9% | Limb weakness | 24 / 46.2% |
| Loss of empathy | 0 / 0% | Weight loss | 2 / 3.9% |
| Insomnia | 0 / 0% | Pain | 18 / 34.6% |
| Hypersomnia | 2 / 3.9% | Neuropathy (sensory / motor) | 34 / 65.4% |
| None of these | 10 / 19.2% |  |  |
| Answered | 52 |  |  |
